# The Effect of Ostracism and Optional Participation on the Evolution of Cooperation in the Voluntary Public Goods Game

**DOI:** 10.1371/journal.pone.0108423

**Published:** 2014-09-25

**Authors:** Mayuko Nakamaru, Akira Yokoyama

**Affiliations:** Tokyo Institute of Technology, O-okayama, Meguro, Tokyo, Japan; University of Maribor, Slovenia

## Abstract

Not only animals, plants and microbes but also humans cooperate in groups. The evolution of cooperation in a group is an evolutionary puzzle, because defectors always obtain a higher benefit than cooperators. When people participate in a group, they evaluate group member’s reputations and then decide whether to participate in it. In some groups, membership is open to all who are willing to participate in the group. In other groups, a candidate is excluded from membership if group members regard the candidate’s reputation as bad. We developed an evolutionary game model and investigated how participation in groups and ostracism influence the evolution of cooperation in groups when group members play the voluntary public goods game, by means of computer simulation. When group membership is open to all candidates and those candidates can decide whether to participate in a group, cooperation cannot be sustainable. However, cooperation is sustainable when a candidate cannot be a member unless all group members admit them to membership. Therefore, it is not participation in a group but rather ostracism, which functions as costless punishment on defectors, that is essential to sustain cooperation in the voluntary public goods game.

## Introduction

Different types of collective action are observed among many species, such as microbe organisms, insects, fish, birds, and mammals including humans. Fish schools, insect swarms, flocks or herds of many species move together following simple rules such as imitating their neighbors’ behaviors or aligning with them, without complex signaling systems or high cognitive abilities [1], [2]. Even though humans have highly developed cognitive abilities, theoretical research shows that the movement of a human mass or crowd can be explained by simple rules [3]. To maintain collective action in some species such as microbes, social insects, and humans, cooperation within groups is required [4], [5]. Cooperators invest their time, energy or money to maintain a group, while free riders do nothing but receive the benefit produced by cooperators. If free riders increase in a group, collective action collapses.

The evolution of cooperation is an unsolved problem from the viewpoint of evolution and social science. Recent theoretical studies show several mechanisms at work to promote the evolution of cooperation: kin selection [6], group selection [7], [8], network structure [9]–[12], direct reciprocity [13], indirect reciprocity [14]–[18], punishment [19]–[24], and rewarding cooperators [25]–[27]. The public goods game (PPG) is the basic model used to describe the difficulty of maintaining cooperation in a group.

Theoretical and experimental studies using the public goods game have shown that cooperation cannot be sustainable in groups if members are selected randomly from the population [28]. A spatially structured population, such as a lattice model or other types of social network models, basically promotes the evolution of cooperation because the spatial structure causes cooperators to gather closely together; cooperators can interact with neighboring cooperators and thus avoid being cheated by free riders [9]–[11]. This assortative interaction promotes evolution of cooperation in groups of not only humans [28]–[32] but also of microbes [33], [34], because individuals choose assortative individuals as group members. However, in the case of human society, if the rich contribute to collective action more than the poor proportional to their wealth, assortative interaction or homophily, in which players in the same wealth class interact assortatively, does not promote the evolution of cooperation [35].

In our daily lives, who can be chosen as a new group member or how a new member decides to participate in a group is important to maintain cooperation or friendship within the group, such as a club [14]. Experimental study shows that when adolescent school students form groups, members can come to resemble each other after they have become a group, rather than by choosing assortative members [36]. This indicates that the premise that groups are formed assortatively is not always a proper assumption. Another example is the screening of a secret organization, such as Freemasonry. That organization screens those who apply for membership before deciding whether to admit them as members.

How group members are selected and whether an individual participates in a group are essential for collective action. Theoretical studies of animal behavior have addressed the evolutionary advantage of group forming or joining a group in resource allocation and food exploitation [37], [38]. However, these studies did not answer the question. Previous studies about evolutionary game theory have shown that nonparticipation in the public goods game promotes the evolution of cooperation [39]–[41] and coevolution of cooperation and punishment [22]. However, these studies assumed that nonparticipants never participate in the public goods game, regardless of group members. Theoretical studies have investigated whether defector exclusion or ostracism from groups promotes the evolution of cooperation [42]–[45]. If defectors suffer the cost of exclusion from the group because that exclusion causes them some damage in common pool resource use, cooperators increase in number and common-resource management can be sustainable [43], [44]. Players called excluders who cooperate and exclude defectors from the group can solve the problem of second-order free riders, in which punishing cooperators diminish after the population is dominated by pure cooperators and punishing cooperators, when excluders find and exclude defectors by paying a cost; excluded defectors cannot get benefits from cooperators [45].

Reputation can be used to choose members or groups. The following are evolutionary game studies about reputation. Theoretical studies of the evolution of indirect reciprocity investigate the evolution of cooperation in the two-player donation game in which a player cooperates with an opponent whose reputation is good; otherwise, the player does not cooperate [15]. Recent studies have focused on the effect of the assessment rule, which determines who is good or bad, on the evolution of cooperation. Other works investigate if group favoritism can be proven by indirect reciprocity in the two-player donation game [16]–[18]. Suzuki and Akiyama (2005) applied the framework of Nowak and Sigmund [15] to the *n*-person prisoner’s dilemma game in which more than two players participate, and each player can choose cooperation when the average reputation of the other members is equal to or higher than the threshold of the focal player [46]. There are studies in which players choose a partner without using reputation as a cue. For example, Aktipis (2004) used the repeated prisoner’s dilemma game and showed that cooperation can be established when cooperators can run away from defectors in a spatially structured population [47]. Chiang (2008) showed that partner choice based on payoff reinforcements from past interaction promotes the evolution of fairness between two players in the ultimatum game [48].

In this paper, we focus on how individuals decide to participate in a group and/or how the group members decide to exclude some individuals from membership when they contribute to collective action, using the voluntary public goods game. The decision-making of a player who chooses a partner in a dyadic interaction is different from that of a player who participates in a group consisting of more than two players, or who excludes individuals from membership. The reasons for this are as follows. When we want to participate in a group, we may evaluate all members or representative members of the group. We decide to (or not to) participate in the group after we observe the best (or worst) member in the group and then regard the group as good (or bad). Similarly, when an individual wants to participate in the group, group members evaluate the individual. In some groups, when all members agree that a candidate is good, that individual is allowed to participate in the group. In other groups, when at least one member decides that a candidate is good, that individual is allowed to join. Therefore, we investigate what types of decision-making of groups or individuals influence the evolution of cooperation in the voluntary public goods game, based on the framework of Nowak and Sigmund [15]. When members of a group exclude some individuals from membership, this results in ostracism and is interpreted as costless punishment. Therefore we investigate the effects of ostracism as costless punishment, and the effects of participation in the group, on the evolution of cooperation in the voluntary public goods game.

## Model

The population has *N* number of players. Each player has three evolutionary traits, one concerning the contribution to the public goods game and two concerning the threshold of decision-making (*k_pa_* and *k_ex_*). There are two types of traits regarding the contribution to the PGG, a cooperator (C) and defector (D). Trait *k_pa_* is the threshold of decision-making when participating in a group. Trait *k_ex_* is the threshold of decision-making when choosing a new member. In addition to these three traits, each player has his own reputation, called image score (*s*) [15]. If a player cooperates, his image score increases by one unit; if the player does not cooperate, his image score decreases one unit. Therefore, a high image score means that the player invests substantially in the PGG, and a low image score means that the player does not invest much. It is assumed that every player knows the reputations of all players. Initially, *s* of each player is zero.

### The following outlines the flow during one unit of time

(i) *N* players in a population are randomly divided into *N*/*m* groups. Each group has *m* players on average, who are called candidates for group members.

(ii) The candidates can be members of the group, according to decision-making called the peer selection rule, which is dependent on the traits (*k_pa_*, *k_ex_*) and image score, *s*. The peer selection rule consists of two conditions: the participation condition and the exclusion condition. In the participation condition, each candidate decides to participate in the group if the group reputation, *S_g_*, is equal to or higher than *k_pa_* (*S_g_* ≥ *k_pa_*). We assume that the group reputation is based on its member’s image scores in a group. We define four types of group reputation: Average *S_g_*, Median *S_g_*, Maximum *S_g_* and Minimum *S_g_*. Average *S_g_* is defined as the average value of all candidate reputations in the group; Median *S_g_*, the median value of all candidate reputations in the group; Maximum *S_g_*, the maximum value for all candidate reputations in the group; Minimum *S_g_*, the minimum value for all candidate reputations in the group. Maximum *S_g_* can be interpreted as that a candidate decides to participate in the group if the best reputation in the group is equal to or higher than the candidate’s threshold (Maximum *S_g_* ≥ *k_pa_*). This assumption corresponds to the situation that people decide to join the group because the group has such a good player. Minimum *S_g_* can be interpreted as that a candidate decides to remain in the group if the worst reputation in the group is equal to or higher than the candidate’s threshold (Minimum *S_g_* ≥ *k_pa_*): if all reputations in the group do not meet the threshold of a candidate, the candidate never joins the group. This assumption describes the situation in which people decide not to join a group because it has a very bad player.

In the exclusion condition, each group excludes some candidates from membership if a candidate’s image score (*s*) is less than the group criterion, *K_g_* (*s*<*K_g_*). We define four types of group criteria: Average *K_g_*, Median *K_g_*, Maximum *K_g_* and Minimum *K_g_*. Average *K_g_* is the average of *k_ex_* in the group; Median *K*_g_, the median for *k*_ex_ in the group; Maximum *K_g_*, the maximum for *k_ex_* in the group; Minimum *K_g_*, the minimum for *k_ex_* in the group. Average or Median *K_g_* means that candidates are selected as group members according to the group average or median of *k_ex_*. Maximum *K_g_* can be interpreted as that a candidate can be a member if the most stringent player in the group accepts the candidate, or if all players in the group accept or vote in favor of the candidate. Minimum *K_g_* can be interpreted as that a candidate can be a member if the most lax player in the group accepts or votes in favor of the candidate.

There are four kinds of peer selection rules, participation selection, exclusion selection, participation-exclusion selection with the same threshold (or same PE selection), and participation-exclusion selection with different thresholds (or different PE selection). The participation selection consists of the participation condition; the exclusion selection, the exclusion condition; the same PE selection, both the participation and exclusion conditions with *k_pa_* = *k_ex_*; the different PE selection, both the participation and exclusion conditions with *k_pa_* ≠ *k_ex_*. In the same and different PE selections, the participation and exclusion conditions occur simultaneously: a candidate can be a member if the reputation level (*s*) of the candidate is higher than or equal to *K_g_* (*s* ≥ *K_g_*) and *S_g_* is higher than or equal to *k_pa_* of the candidate (*S_g_* ≥ *k_pa_*). Therefore, will investigate the effect of 16 types of peer selection rules on the evolution of cooperation in the voluntary PGG. That is, the participation selection with one of four group reputations, exclusion selection with one of four group criteria, the same PE selection with one of four criteria (Average *K_g_* and Average *S_g_*, Median *K_g_* and Median *S_g_*, Maximum *K_g_* and Maximum *S_g_*, Minimum *K_g_* and Minimum *S_g_*), and the different PE selection with one of four criteria (Average *K_g_* and Average *S_g_*, Median *K_g_* and Median *S_g_*, Maximum *K_g_* and Maximum *S_g_*, Minimum *K_g_* and Minimum *S_g_*). Later, we investigate three other possible types of peer selection rule.

(iii) Members in each group play the PGG once in which a cooperator (C) contributes to public goods and a defector (D) does not contribute at all. The payoffs for C (*π^C^*) and D (*π^D^*) in the group are defined as *π^C^*(*n_c_*) = *bxn_c_*/*n* − *x* and *π^D^*(*n_c_*) = *bxn_c_*/*n*, in which *n* (*n* ≥2) is the number of group members and *n_c_* (0≤ *n_c_* ≤ *n*) is the number of Cs in the group; *b* is the benefit factor produced by cooperators and *x* is the contribution to the public goods or the cost of cooperation. Here, two conditions, *π^D^*(*n_c_*)>*π^C^*(*n_c_* +1) and *π^D^*(*n*)>*π^C^*(0), meet the definition of social dilemma. We assume 1< *b* <2, because *n* = 2 is the minimum group size after peer selection. The payoff for the players who are excluded from membership is zero, since they do not play the PGG. If the group only consists of one member, the payoff for that player is zero. Therefore, if defectors participate in groups, their payoff is higher than zero if the group has one or more cooperator. If cooperators participate in groups, their payoff is higher than zero if *n_c_*/*n* >1/*b*. Because the value of *b* is between 1 and 2, *n_c_*/*n* should be higher than 0.5 at least, if cooperators within groups receive benefits from participating in them. Otherwise, it is better for cooperators not to participate in groups than to do so.

(iv) The image score (*s*) of each player is given after playing the PGG. The image score of a player increases by one unit if the player cooperates or invests in the PGG, and decreases by one unit if the player is a defector. If a player is eliminated from the group according to the peer selection rule and does not play the PGG, his image score does not change.

After steps (i)–(iv) are repeated *h* times, the total score of each player, defined as his accumulated payoff during one generation, is obtained. Each player updates his traits to those of a player called A, in proportion to the total score of player A over the sum of the total scores of all players. This is interpreted as social learning. Each player changes his traits randomly with probability *μ*, which corresponds to a probability of mutation in evolutionary game theory. This algorithm corresponds to natural selection from the standpoint of evolution. Then one generation, consisting of *h* units of time, ends.

In the next generation, image score and the total score are reset to zero. We analyzed this model through 10,000 generations by computer simulation. Baselines for the three parameters are *N* = 100, *m* = 5, *h* = 10, *x* = 1, and *μ = *0.005. The values of *k_pa_* or *k_ex_* are uniform random numbers from integers between −6 and +6 at the beginning of the simulation run and when a mutation occurs. Initially, the image score (*s*) of each player is zero and is constrained between −5 and +5 [15]. The initial population consists of defectors, to investigate if cooperators can invade a population dominated by defectors.

## Results

In the baseline model, in which all members of each group can participate in the PGG because the peer selection rule is not implemented, cooperation never evolves, regardless of *N*, *h*, *m* and *b*. This is because the payoff of D is always higher than that of C. We next introduced the peer selection rules to the baseline model to determine how each of the peer selection rules influenced the evolutionary outcomes.

**Figure 1:**
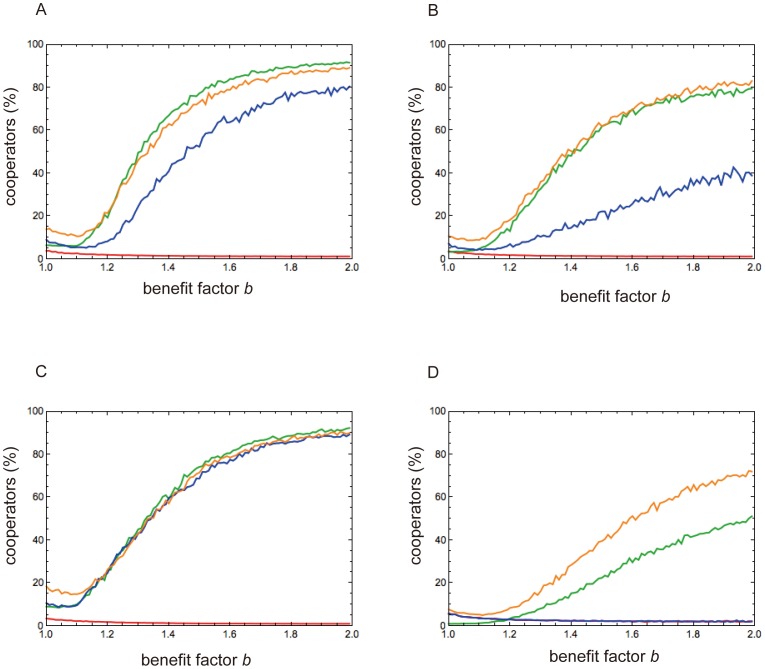
Simulation outcomes of baseline model with peer selections. Vertical axis is average percentage of cooperators in entire population over 100 runs, in each of which 10,000 generations were simulated. Horizontal axis represents benefit factor for cooperation *b*. (A) is for Average criterion; (B) Median criterion; (C) Maximum criterion; (D) Minimum criterion. Red line represents the participation selection, green line the exclusion selection, blue line the same PE selection, and orange line the different PE selection. In (D), red and blue lines overlap. Other parameters are *N* = 100, *m* = 5, *h* = 10, and *μ* = 0.005.

Figures 1 and S1–S5 show the evolutionary simulation outcomes for the four criteria and four peer selection rules. Figure S1 shows that a nonparticipating defector is in the majority in any selection with any criterion in which benefit factor *b* is low. As *b* increases, the number of participants increases; a higher *b* increases the payoff of the players when they join the group and play the PGG. Naturally, the number of nonparticipating cooperators declines when *b* increases. As a result, the number of cooperators, which is the sum of participating and nonparticipating cooperators, decreases as *b* slightly increases when *b* is small and is less than ∼1.1 (Fig. S1). In the participation selection, although *b* is high, cooperation never evolves regardless of criterion type (Fig. 1). This result indicates that the existence of nonparticipants does not promote the evolution of cooperation, if players can evaluate a group and decide whether to participate in it. This is contrary to previous studies, in which nonparticipants who never evaluated groups promoted such evolution. Cooperation especially evolves in the exclusion selection and the different PE selection, with average or maximum criteria (Fig. 1A and C). Figure 1D indicates that the minimum criterion in any peer selection rule does not increase the cooperation rate more than other criteria. Simulation outcomes in the average criterion are different from the median one, but similar to the maximum criterion. This implies that the distribution of image score or thresholds after selection is not a normal or uniform one, even though thresholds are randomly determined to be uniform and the image score of all players is zero at the beginning of simulation. In the following, we explain why bursts of defectors and nonparticipants occur in Figs. S2–S5 D, E, G, H, K, and L. When mutation produces many defectors by chance, the image score of a player can be less than *K_g_* (Figs. S2–5 D–F). As a result, many players are excluded from membership and payoffs are low. If cooperators can join the group and they dominate the group, they can obtain high payoffs. Then, the frequency of cooperators increases again. The same mechanism is depicted in Figs. S2–5 G–N. In conclusion, ostracism promotes the evolution of cooperation if the group excludes some individuals who are regarded as bad, either by all members or by the average.

Now, we discuss why the exclusion selection promotes the evolution of cooperation but the participation selection does not. In the latter selection, players can decide to participate in a group. Participating defectors obtain more benefits than nonparticipating ones with higher *b*. Then, defectors want to join the PGG, and the number of nonparticipating defectors is high with low *b* and the number of participating ones is high with high *b.* Since the participation selection has no mechanism for excluding defectors from membership, defectors come to dominate groups. Consequently, cooperators do not obtain high payoffs although they participate in the group, so their numbers diminish. For example, when Maximum *S_g_* is used in the participation selection, other members except the one with the maximum image score in the group do not have an incentive to choose cooperation, and then do not have a high image score. Even if the group has one member with a very good reputation and others with a bad reputation, a candidate decides to participate in the group. The members with bad reputation, many of whom are defectors, receive a benefit from a newcomer if he is a cooperator. As a result, cooperation never evolves. Since the exclusion selection excludes defectors from membership, cooperators have high payoffs when they join groups and play the PGG. As a result, cooperators dominate groups, especially with higher *b* (Fig. S1).

The following shows why the maximum criterion promotes cooperation but the minimum one never does so in the exclusion selection. The maximum criterion in that selection means that a candidate cannot become a group member unless all players in the group accept him. This is very strict exclusion or ostracism and consequently, defectors are excluded from membership and cooperation can then evolve. The minimum criterion in the exclusion selection means that when one player in a group accepts a candidate, that candidate can obtain membership. This corresponds to the situation in which only one vote is needed to become a member. This means that defectors can obtain a membership more easily with the minimum criterion than with the maximum one (Fig. S1 G and H; the exclusion selection in Fig. S6 C and D). As a result, the minimum criterion in the exclusion selection does not promote cooperation.

Cooperation evolves in the different PE selection more than in the same PE selection. The participation and exclusion conditions are independent of each other in the different PE selection. Then, the exclusion condition in that selection works as efficiently as the exclusion selection does. If the threshold of the participation condition (*k_pa_*) is the same as that of the exclusion condition (*k_ex_)* in the same PE selection, the exclusion condition in the latter selection does not work efficiently because the participation condition hinders the exclusion condition in the same PE selection.

**Figure 2:**
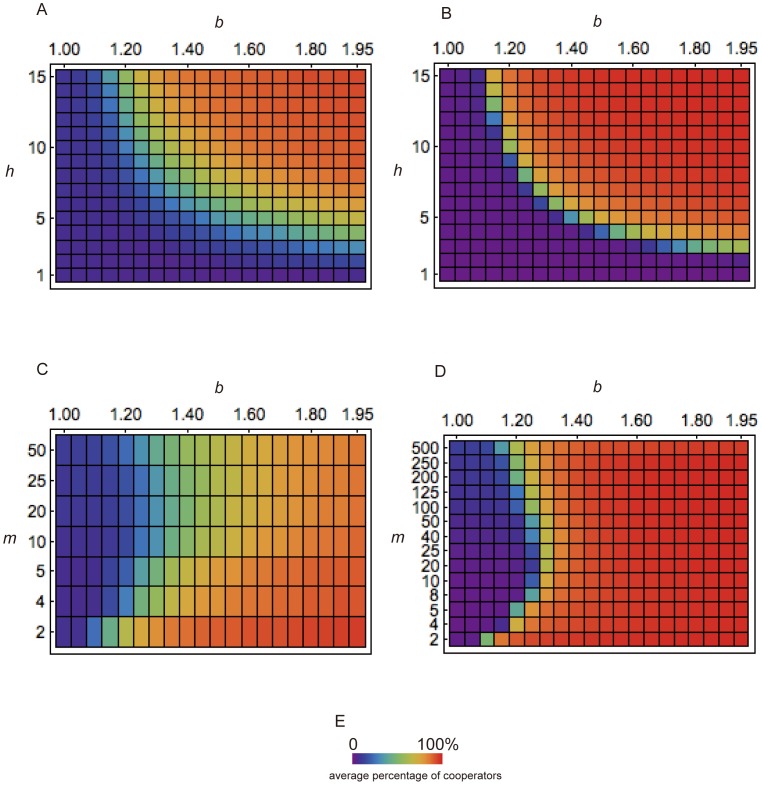
Parameter dependence when exclusion selection with Average *K_g_* is applied. Shown is the average percentage of cooperators in the entire population over 100 runs, in each of which 10,000 generations were simulated. (A) and (B) show the effect of parameters *h* and *b* on simulation outcomes when *m* = 5. (C) and (D) show the effect of parameters *m* and *b* on simulation outcomes when *h* = 10. (A) and (C) are for *N* = 100, and (B) and (D) for *N* = 1,000. (E) presents the relationship between percentage and color in all graphs. The other parameter is *μ* = 0.005.

Figures 2, S7 and S8 show the effect of *h, m* and *b* on the evolution of cooperation with *N* = 100 and *N* = 1,000. When *h* and *b* are high, cooperation is favored. The result for *N* = 1,000 is basically the same as for *N* = 100. However, the evolved cooperation level is higher with *N* = 1,000 than *N* = 100 when *b* and *h* are high, and that level is lower with *N* = 1,000 than *N* = 100 when *b* and *h* are low. This is because stochasticity influences evolutionary dynamics more with smaller *N*. Figure 2 also shows that smaller *m* promotes the evolution of cooperation. When *m* is higher higher than 20, the larger group size appears to promote cooperation. However, because the net group size after peer selection *m*’ is smaller than *m* (Fig. S6) and the ratio of nonparticipants increases, we cannot conclude that large group size promotes the evolution of cooperation under peer selection.

**Figure 3:**
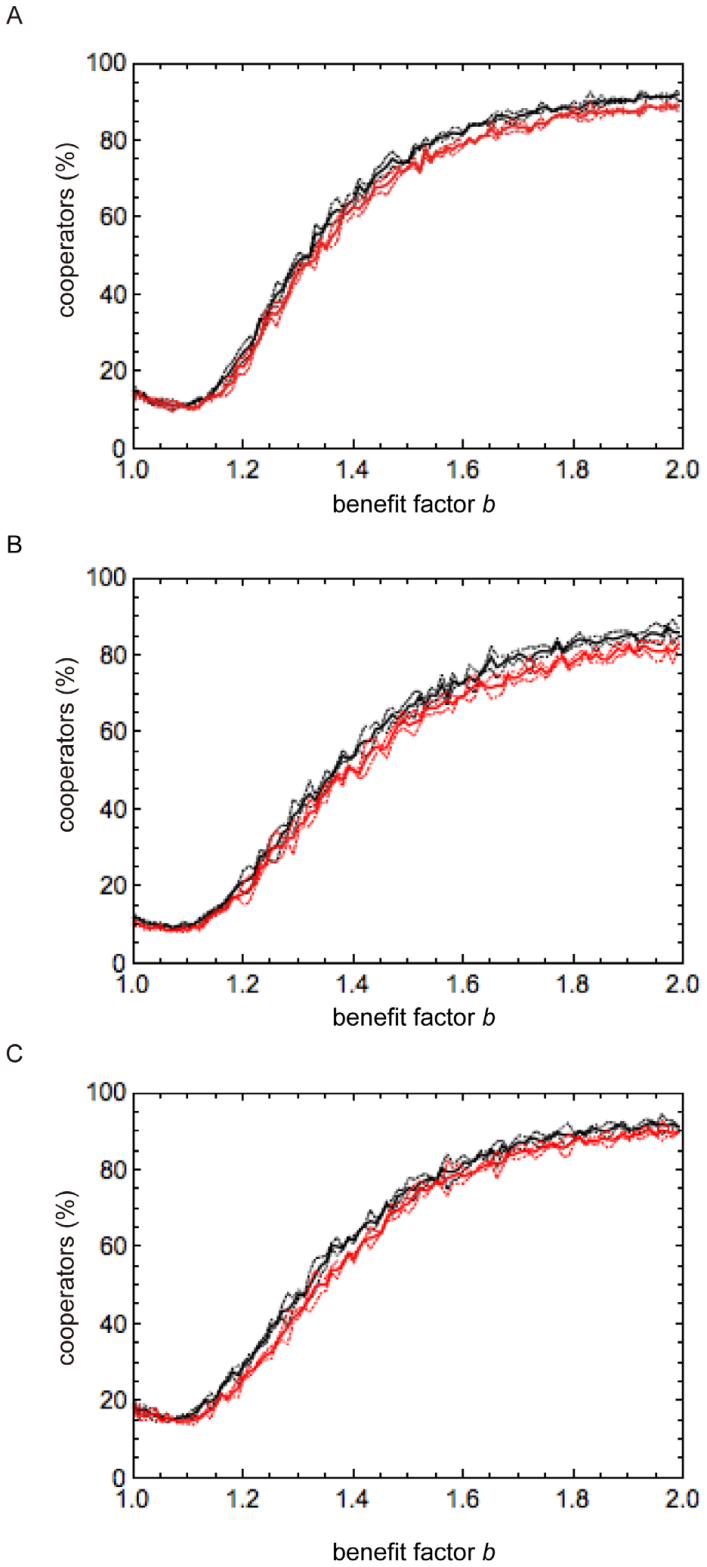
Comparison for the different PE selection between the same and different criteria. Vertical axis is the average percentage of cooperators in the entire population over 100 runs, in each of which 10,000 generations were simulated. Horizontal axis represents benefit factor for cooperation *b*. (A) Black line represents simulation outcomes when the different PE selection with Average *K_g_* and Minimum *S_g_* is used, and red line when the different PE selection with Average *K_g_* and Average *S_g_* is used. (B) Black line represents simulation outcomes when the different PE selection with Median *K_g_* and Minimum *S_g_* is used, and red line when the different PE selection with Median *K_g_* and Median *S_g_* is used. (C) Black line represents simulation outcomes when the different PE selection with Maximum *K_g_* and Minimum *S_g_* is used, and red line when the different PE selection with Maximum *K_g_* and Maximum *S_g_* is used. Dashed lines indicate double standard deviation (95% confidence interval). The other parameters are *N* = 100, *m* = 5, *h* = 10, and *μ* = 0.005.

If the group can properly exclude defectors from membership, members may obtain a high benefit despite the application of Minimum *S_g_* in the participation condition. In the minimum criterion, the cooperation rate in the different PE selection is much higher than that in the exclusion selection (Fig. 1D). However, for other criteria, cooperation rates in both the exclusion selection and different PE selection are nearly the same (Fig. 1A, B and C). This result implies that the combination of the exclusion condition with other criteria (maximum, average, or medium *K*_g_) and the participation condition with Minimum *S*_g_ promote the evolution of cooperation in the different PE selection. Figure 3 shows that cooperation is slightly more favored when Maximum, Average or Medium *K*_g_ is used in the exclusion condition and Minimum *S*_g_ is used in the participation condition, relative to when the same criterion is used in both the participation and exclusion conditions, especially when the benefit from the PGG (*b*) is high. However, the cooperation rate in the different PE selection with Maximum (or Average) *K*_g_ and Minimum *S*_g_ is nearly the same as in the exclusion selection with Maximum (or Average) *K*_g_. Therefore, if both the exclusion and participation conditions are needed, cooperation is established when two decision-making principles are obeyed: (i) if a candidate wishes to participate in a group, they should see if the worst person or all persons can satisfy that candidate’s criterion and then decide to participate in it; and (ii) if all players in a group accept a candidate, that candidate can become a member.

## Discussion

We investigated the effect of optional participation and ostracism on the evolution of cooperation in contributing to collective action, which is described by the voluntary PGG based on the framework of Nowak and Sigmund [15]. We showed that the existence of nonparticipants does not promote cooperation when players evaluate others in a group that they are deciding whether to join. When players in a group evaluate others and decide whether to exclude them from membership, cooperation can evolve. This indicates that exclusion from groups, which can be interpreted as ostracism and functions as costless punishment, promotes the evolution of cooperation. This is especially so when a candidate for group member is excluded unanimously, or when a candidate is excluded because their reputation or image score is below the average of thresholds for the group; then, the cooperation rate can be high through evolution.

Our results indicate that if group membership is open to all who are willing to participate in it, group cooperation is not sustainable. The exclusion condition, in which group members exclude some candidates from membership if their reputation does not satisfy the threshold, is essential from the standpoint of sustaining group cooperation. However, in daily life, group members cannot exclude individuals who are unwilling to participate in the group; thus, PE selection consisting of both the participation and exclusion conditions is more realistic than the exclusion selection. Examples of institutions that require PE selection are a rotating savings and credit association (ROSCA) and microcredit [31], [49], [50]. In a ROSCA, which is an informal institution that exists worldwide, a group consisting of *y* members is formed and members invest their money in a pool at each meeting. Then, one member can receive the fund by lot or bidding. After *y* meetings, all members can obtain the fund. If a group has defaulters who stop investing money after receiving the fund, the ROSCA collapses. Therefore when forming a group, its members must properly choose new members or individuals have to choose a proper group. Otherwise, people lose their investments. On Sado Island, offshore of central Japan, some older people still enjoy participating in ROSCAs. On January 25, 2013, my coworker and I interviewed a woman who joined a ROSCA with seven or eight other elderly women. She stated that a person who wanted to participate in her ROSCA could not be a member unless members admitted that person to membership unanimously. This corresponds to Maximum *K_g_* in the exclusion condition of our model. A ROSCA candidate may use Minimum *S_g_* because the person wants to join the ROSCA if all members’ reputations satisfy his threshold. Therefore, the different PE selection with Maximum (or Average) *K*_g_ and Minimum *S*_g_ may more accurately describe reality. To verify how to choose a new member and a group in reality, we must do more field research. Microcredit, which originated from a ROSCA, is another example with the same characteristics [31]. A microfinance bank lends money to a group and then if some of its members do not repay this money, other members cannot make or receive any more loans.

Our results indicate that cooperation can be better sustained in a group when individuals use different thresholds for participation and exclusion (*k_pa_* ≠ *k_ex_*), more so than when such individuals use the same threshold for participation and exclusion (*k_pa_* = *k_ex_*) in the PE selection. We also showed that “Minimum *S_g_* and Maximum *K_g_*” or “Minimum *S_g_* and Average *K_g_*” should be used to create a high cooperation rate. The experimental studies showed that exclusion promotes cooperation in the PPG when players can cast a vote for excluding others from the group based on their past behavior, gossip, or reputation [51], [52], This is partially supported by our results concerning the exclusion selection. To further verify our conclusion, experimental work should be undertaken to help examine people’s decision-making with respect to participation in and exclusion from groups.

Now, we compare our results of participant selection with Hauert *et al*. [40]. They assumed that: (i) the population has three strategies, cooperators, defectors and nonparticipants; and (ii) payoff of the nonparticipants (*σ*) is between 0 and *xb* – *x*, in which *x* is the contribution to the pool [40]. Hauert *et al*. showed that the population is dominated by nonparticipants in equilibrium when 1< *b* <2 and three strategies coexist when *b* >2. We assumed that: (i) there were two strategies, cooperators and defectors, and whether to participate in a group was not determined by an evolutionary trait but by the decision-making of each player; and (ii) *σ = *0. Our result showed that nonparticipating and participating defectors exist in equilibrium when the participant selection is applied. In Fig. S1 A–D, the number of participating defectors increases and that of nonparticipating defectors decreases when *b* increases. This is because the payoff of participating defectors is slightly higher than that of nonparticipating defectors, as the population has a small number of participating cooperators (Fig. S2 A and B; Fig. S3 A and B; Fig. S4 A and B; Fig. S5 A and B). We did not perform simulations for *b* >2, because social dilemma in a group consisting of *n* members (*n* ≥2) is solved if *b*>*n*. In future study, we will investigate the effect of *b* >2 on the evolution of cooperation, but we must check if *b* is less than the group size of all groups in the population during simulations. Even if we had assumed that *σ* was positive, our results would have be different from [40]. This is because players never evaluate a group when they decide whether to join it [40].

We assumed that the reputation (or image score) of nonparticipants did not change. However, the reputation of nonparticipants may decrease by one unit if they are excluded from membership. Or, that of nonparticipants may increase by one unit if they decide not to join a group with bad reputation. Group members may pay a cost of exclusion, and then excluded players may suffer the cost of being excluded [42]–[45]. This suggests that there are other possible ways for defining reputation and costs. In the future, we will elucidate the effects of *σ* and of the costs of exclusion and being excluded on the evolution of group cooperation, and examine how to define the reputation of nonparticipants influences the evolution of group cooperation.

We assumed that individuals screen others based on the history of their behaviors. A field study of an Andean community showed that reputation is related to the contribution to collective action [53]. In our model, reputations of individuals who have not cooperated with defectors are the same as those of individuals who have not cooperated with cooperators. However, some indirect reciprocity studies of dyadic interaction have shown that cooperation is more sustainable when the reputation of an individual who has not cooperated with cooperators can be distinguished from that of one who has not cooperated with defectors than when the reputations of defectors are the same, regardless of whom defectors have not cooperated with [14], [16]. We do not know whether to use second and third order information about the reputation of others when forming a group. We may be confused by complicated information when evaluating people or groups. Some people may not have a sufficiently high cognitive ability to manage such complex information, whereas others can do so. Our model may be appropriate for investigating the effect of participation and exclusion on the evolution of cooperation in groups, because people may not use complex information in forming a group. The effect of cognitive ability on managing reputation is suitable for future study.

In our model, we did not deal with the following situations: (i) people willing to join a particular group can compare more than one group, then choose one group; and (ii) people excluded from membership who look for other groups and then attempt to participate in another group. We will tackle these cases for future research.

## Supporting Information

Figure S1 **Percentage of participating or nonparticipating cooperators and defectors.** Shown is average percentage of participating cooperators (solid blue line), nonparticipating cooperators (dashed blue), participating defectors (solid red), and nonparticipating defectors (dashed red) in the entire population over 100 runs, in each of which 10,000 generations were simulated. Horizontal axis represents benefit factor *b*. (A−D) is for the participation selection, (E−H) the exclusion selection, (I−L) the same PE selection, and (M−P) the different PE selection. (A, E, I, and M) are for Average criterion, (B, F, J and N) for Median criterion, (C, G, K and O) for Maximum criterion, and (D, H, L and P) for Minimum criterion. The other parameters are *N* = 100, *m* = 5, *h* = 10, and *μ* = 0.005.
(TIF)

Figure S2 **Simulation outcomes through 10,000 generations in one run when Average criterion is used.** (A−C) presents results from the participation selection, (D−F) the exclusion selection, (G−J) the same PE selection, and (K−N) the different PE selection. (A, D, G, and K) show the average percentage of participants through generations. Blue line represents participating cooperators and red line participating defectors. (B, E, H, and L) show average percentage of nonparticipants through generations. Light blue line represents nonparticipating cooperators and pink line nonparticipating defectors. (C, J, N) show average *k_pa_* (yellow) and *S_g_* (purple). (F, I, and M) show average *s* (orange) and *K_g_* (green). The average *k_pa_* (or *s*) is the average of *k_pa_*_*_t_* (or *s*_*_t_*) through 10,000 generations, which is the population average of *k_pa_* (or *s*) of each player at the end of the *t*-th generation. *S_g_* (or *K_g_*) is calculated as the average of *S_g_*_*_t_* (or *K_g_*_*_t_*) through 10,000 generations, which is the population average of *S_g_* (or *K_g_*) of each group during *h* units of time at the *t*-th generation. The parameters are *N* = 100, *m* = 5, *h* = 10, *b* = 1.85, and *μ* = 0.005.
(TIF)

Figure S3 **Simulation outcomes through 10,000 generations in one run when Median criterion is used.** See Fig. S2 for detailed information.
(TIF)

Figure S4 **Simulation outcomes through 10,000 generations in one run when Maximum criterion is used.** See Fig. S2 for detailed information.
(TIF)

Figure S5 **Simulation outcomes through 10,000 generations in one run when Minimum criterion is used.** See Fig. S2 for detailed information.
(TIF)

Figure S6 **Average net group size after peer selection.** Horizontal axis is for group size before peer selection, *m*. Vertical axis is for average net group size after peer selection for one hundred simulation runs, in each of which simulation was performed through 10,000 generations. Red point represents the participation selection, green point the exclusion selection, blue point the same PE, and orange point the different PE. (A) is for Average criterion, (B) Median criterion, (C) Maximum criterion, and (D) Minimum criterion. In (D), red and blue points overlap. Dashed lines indicate one standard deviation (68% confidence interval). The other parameters are *h* = 10, *b* = 1.85, *N* = 100, and *μ* = 0.005.
(TIF)

Figure S7 **The effect of parameters** ***m*** **and** ***b*** **on simulation outcomes when** ***h*** **= 10 and** ***N*** **= 100.** Shown is the average percentage of cooperators in the entire population over 100 runs, in each of which 10,000 generations were simulated. (A−D) are for the participation selection, (E−H) the exclusion selection, (I−L) the same PE selection, and (M−P) the different PE selection. (A, E, I and M) are for the Average criterion, (B, F, J and N) the Median criterion, (C, G, K and O) the Maximum criterion, and (D, H, L and P) the Minimum criterion. (Q) presents the relationship between percentage and color in all graphs. (E) is the same as Fig. 2A. The other parameter is *μ* = 0.005.
(TIF)

Figure S8 **The effect of parameters** ***h*** **and** ***b*** **on simulation outcomes when** ***m*** **= 5 and** ***N*** **= 100.** Shown is the average percentage of cooperators in the entire population over 100 runs, in each of which 10,000 generations were simulated. (A−D) are for the participation selection, (E−H) the exclusion selection, (I−L) the same PE selection, and (M−P) the different PE selection. (A, E, I and M) is for the Average criterion, (B, F, J and N) the Median criterion, (C, G, K and O) the Maximum criterion, and (D, H, L and P) the Minimum criterion. (Q) presents the relationship between percentage and color in all graphs. (E) is the same as Fig. 2C. The other parameter is *μ* = 0.005.
(TIF)
