# Supplementary figures and images for: The Effect of Ostracism and Optional Participation on the Evolution of Cooperation in the Voluntary Public Goods Game
Source: PLoS One. 2014 Sep 25;9(9):e108423. doi: 10.1371/journal.pone.0108423 (PMC4177995; doi:10.1371/journal.pone.0108423)

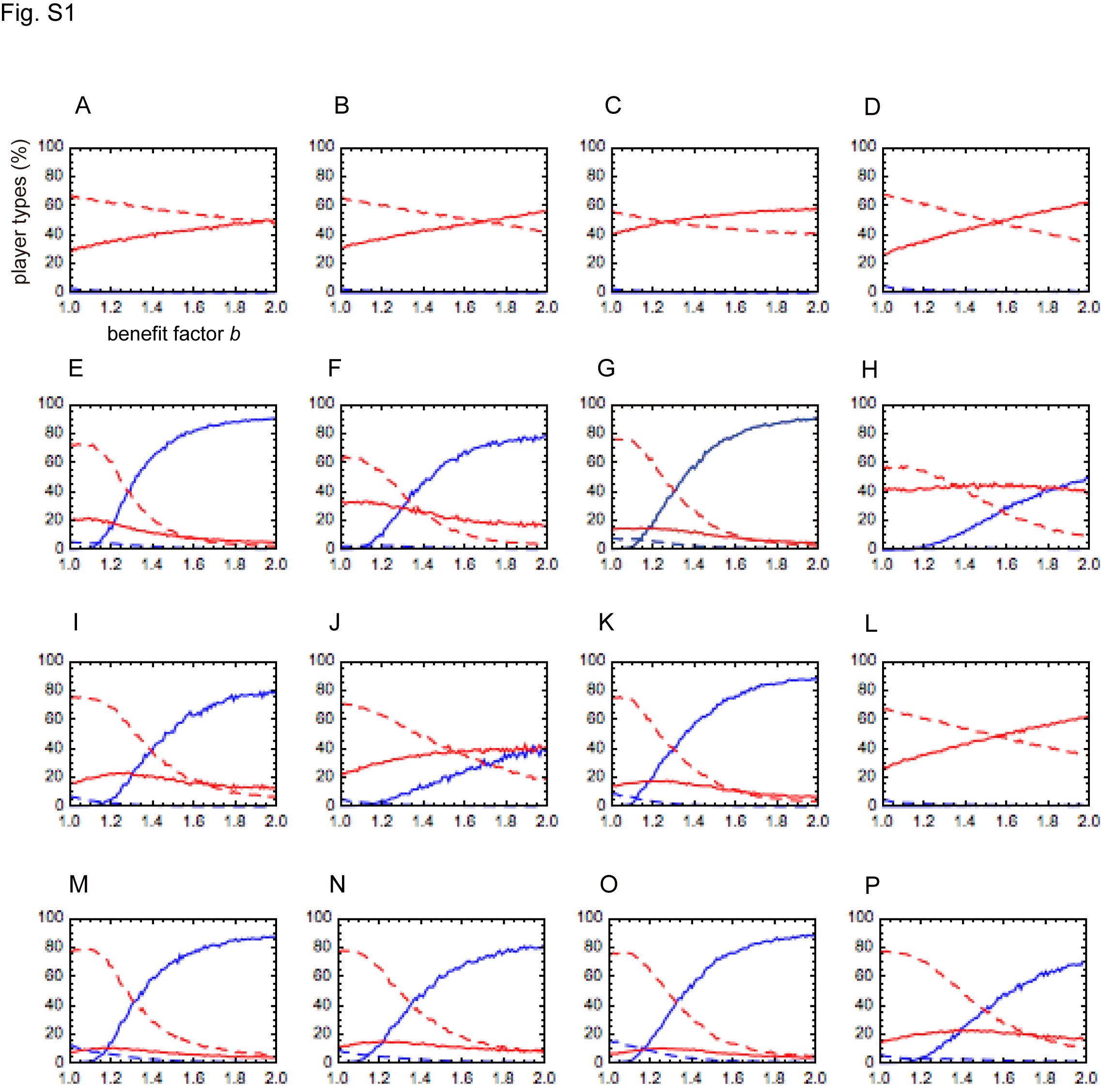

Supplement: Figure S1 — Percentage of participating or nonparticipating cooperators and defectors. Shown is average percentage of participating cooperators (solid blue line), nonparticipating cooperators (dashed blue), participating defectors (solid red), and nonparticipating defectors (dashed red) in the entire population over 100 runs, in each of which 10,000 generations were simulated. Horizontal axis represents benefit factor b. (A−D) is for the participation selection, (E−H) the exclusion selection, (I−L) the same PE selection, and (M−P) the different PE selection. (A, E, I, and M) are for Average criterion, (B, F, J and N) for Median criterion, (C, G, K and O) for Maximum criterion, and (D, H, L and P) for Minimum criterion. The other parameters are N = 100, m = 5, h = 10, and μ = 0.005. (TIF) [file pone.0108423.s001.tif]

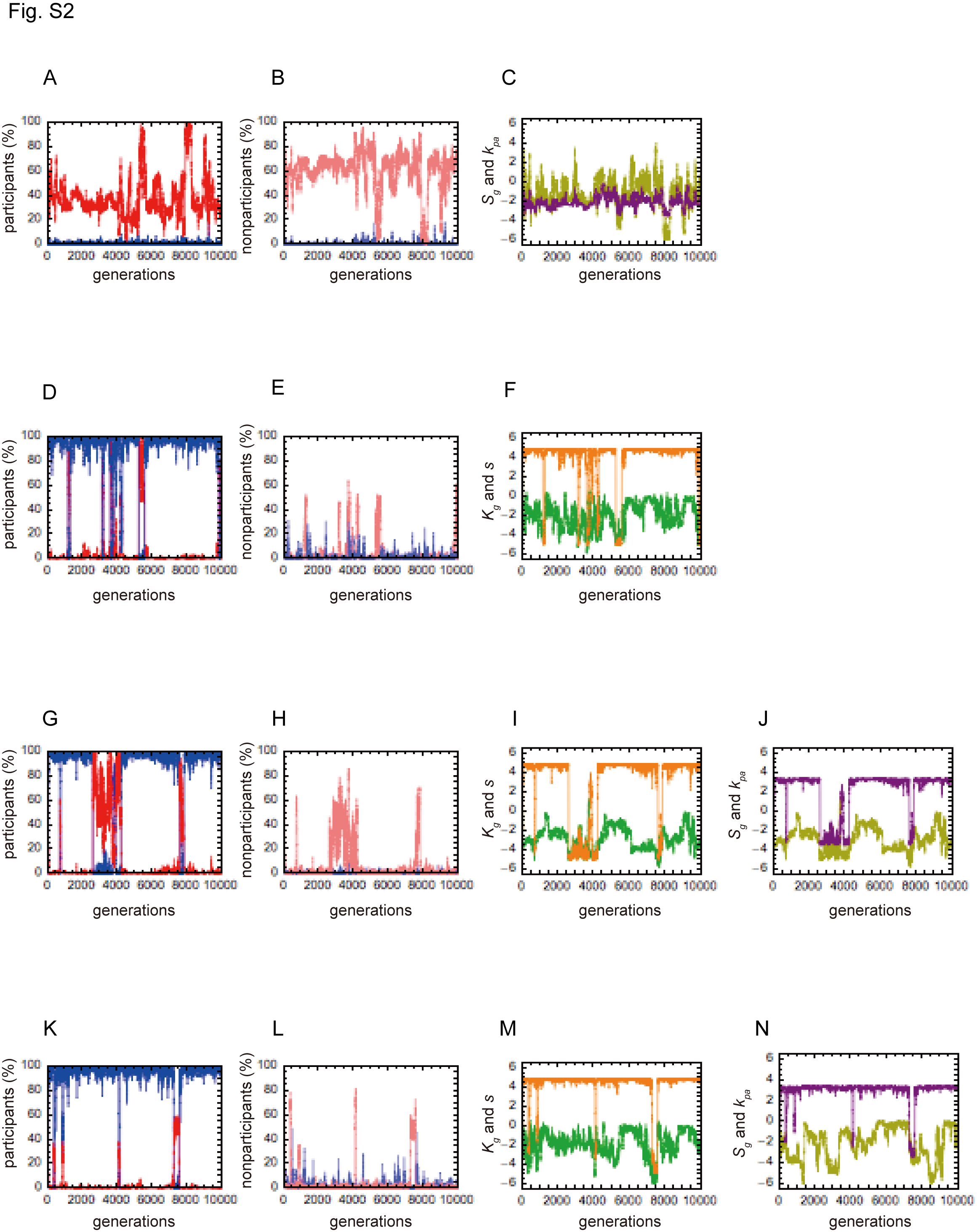

Supplement: Figure S2 — Simulation outcomes through 10,000 generations in one run when Average criterion is used. (A−C) presents results from the participation selection, (D−F) the exclusion selection, (G−J) the same PE selection, and (K−N) the different PE selection. (A, D, G, and K) show the average percentage of participants through generations. Blue line represents participating cooperators and red line participating defectors. (B, E, H, and L) show average percentage of nonparticipants through generations. Light blue line represents nonparticipating cooperators and pink line nonparticipating defectors. (C, J, N) show average kpa (yellow) and Sg (purple). (F, I, and M) show average s (orange) and Kg (green). The average kpa (or s) is the average of kpa_t (or s_t) through 10,000 generations, which is the population average of kpa (or s) of each player at the end of the t-th generation. Sg (or Kg) is calculated as the average of Sg_t (or Kg_t) through 10,000 generations, which is the population average of Sg (or Kg) of each group during h units of time at the t-th generation. The parameters are N = 100, m = 5, h = 10, b = 1.85, and μ = 0.005. (TIF) [file pone.0108423.s002.tif]

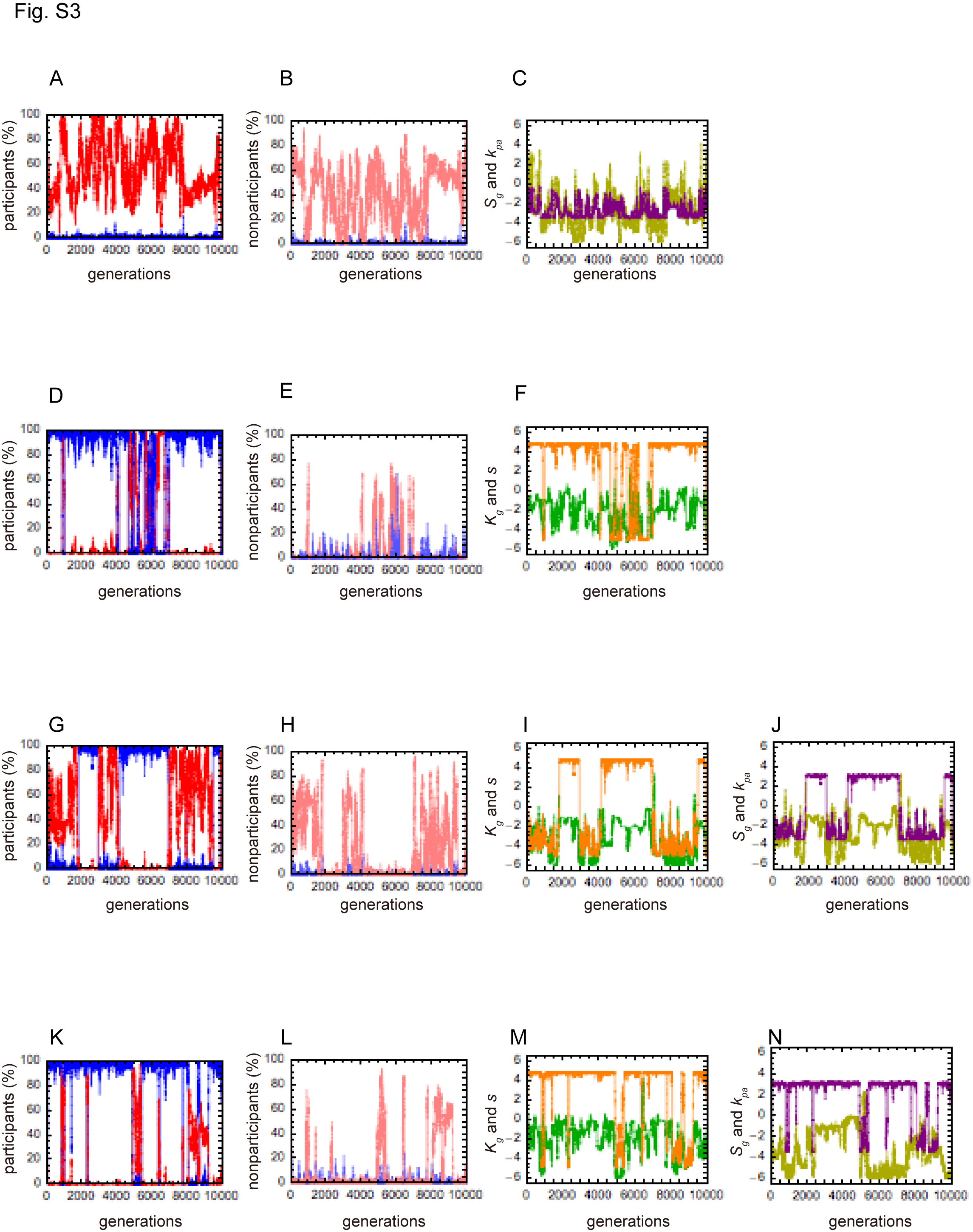

Supplement: Figure S3 — Simulation outcomes through 10,000 generations in one run when Median criterion is used. See Fig. S2 for detailed information. (TIF) [file pone.0108423.s003.tif]

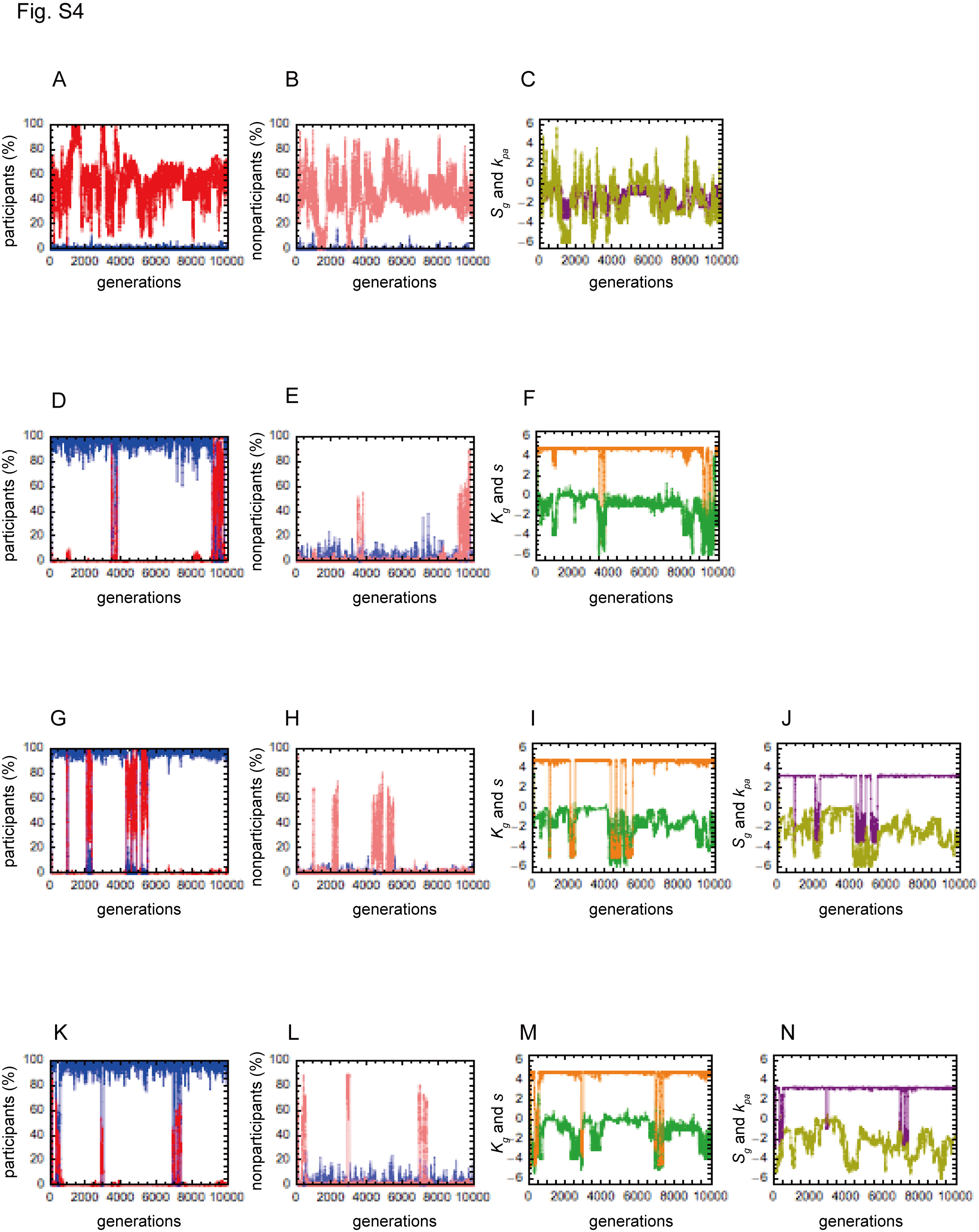

Supplement: Figure S4 — Simulation outcomes through 10,000 generations in one run when Maximum criterion is used. See Fig. S2 for detailed information. (TIF) [file pone.0108423.s004.tif]

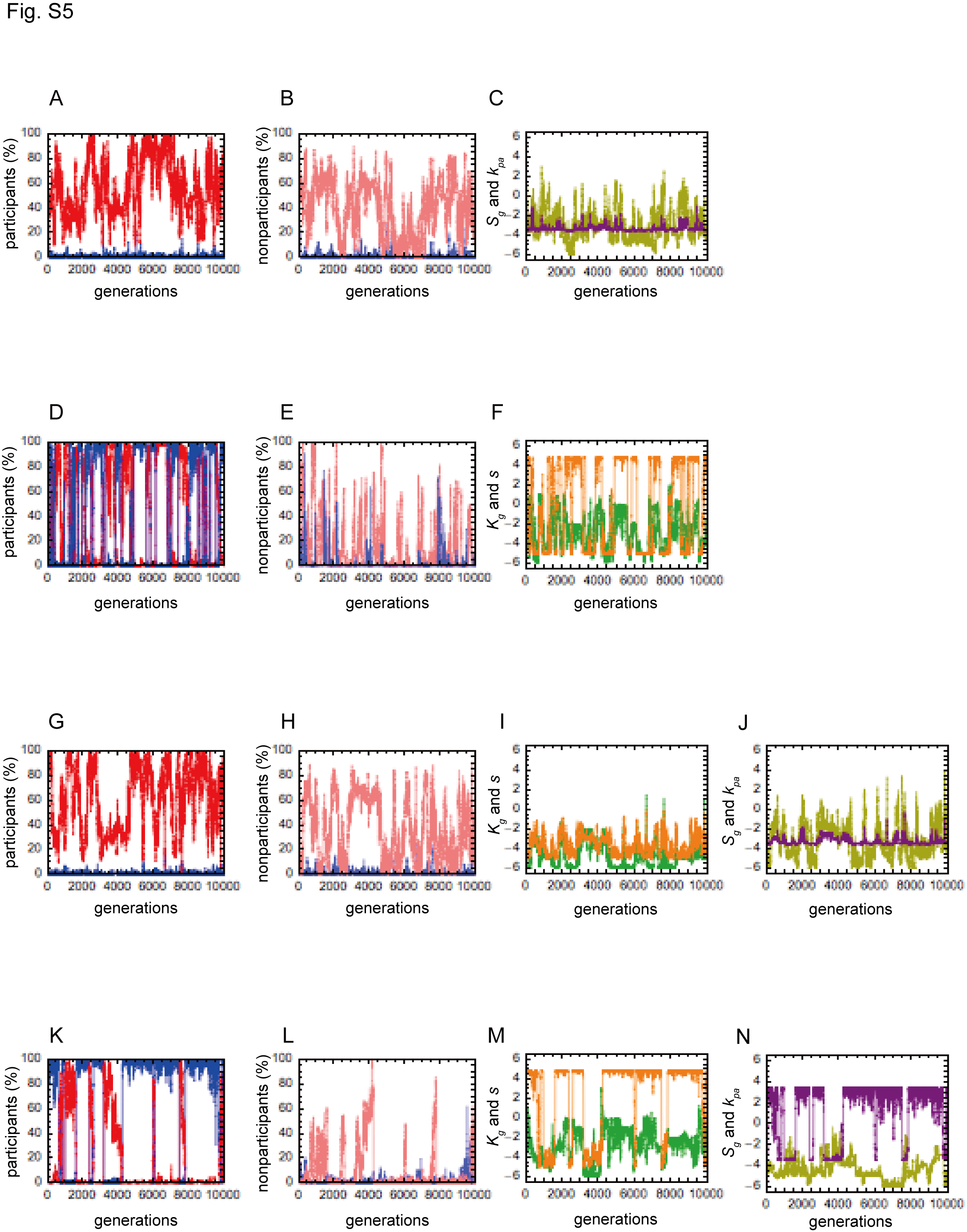

Supplement: Figure S5 — Simulation outcomes through 10,000 generations in one run when Minimum criterion is used. See Fig. S2 for detailed information. (TIF) [file pone.0108423.s005.tif]

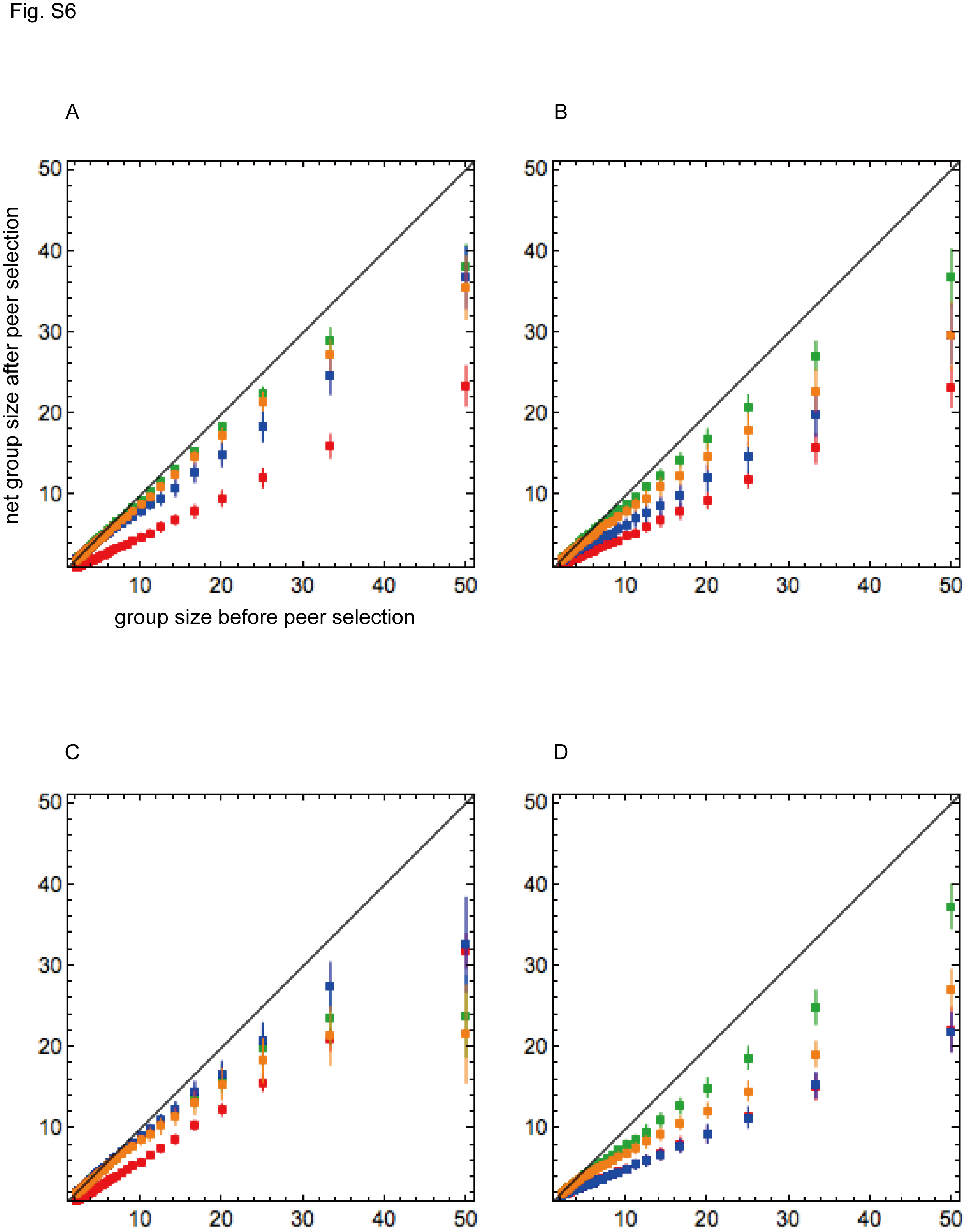

Supplement: Figure S6 — Average net group size after peer selection. Horizontal axis is for group size before peer selection, m. Vertical axis is for average net group size after peer selection for one hundred simulation runs, in each of which simulation was performed through 10,000 generations. Red point represents the participation selection, green point the exclusion selection, blue point the same PE, and orange point the different PE. (A) is for Average criterion, (B) Median criterion, (C) Maximum criterion, and (D) Minimum criterion. In (D), red and blue points overlap. Dashed lines indicate one standard deviation (68% confidence interval). The other parameters are h = 10, b = 1.85, N = 100, and μ = 0.005. (TIF) [file pone.0108423.s006.tif]

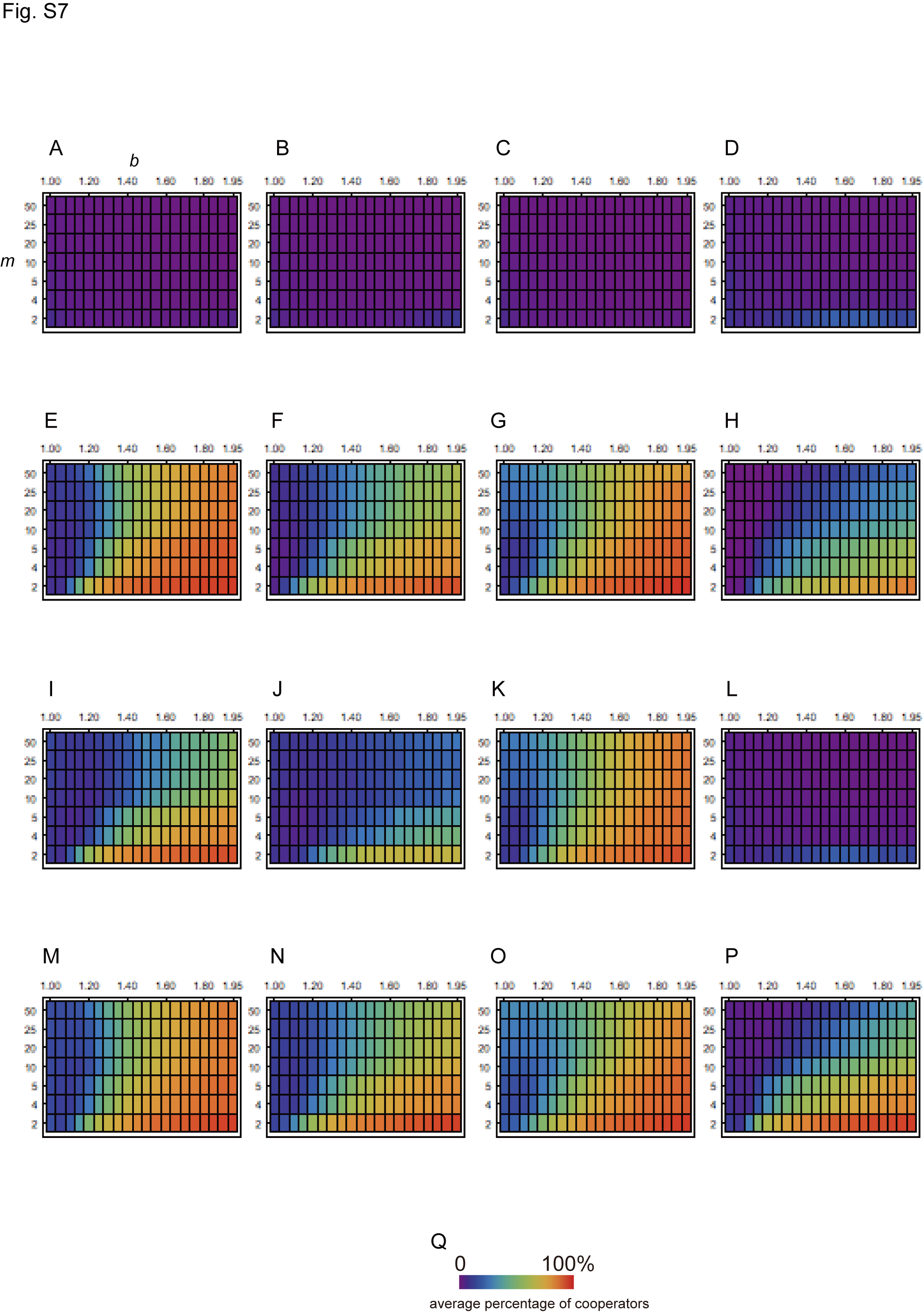

Supplement: Figure S7 — The effect of parameters m and b on simulation outcomes when h = 10 and N = 100. Shown is the average percentage of cooperators in the entire population over 100 runs, in each of which 10,000 generations were simulated. (A−D) are for the participation selection, (E−H) the exclusion selection, (I−L) the same PE selection, and (M−P) the different PE selection. (A, E, I and M) are for the Average criterion, (B, F, J and N) the Median criterion, (C, G, K and O) the Maximum criterion, and (D, H, L and P) the Minimum criterion. (Q) presents the relationship between percentage and color in all graphs. (E) is the same as Fig. 2A. The other parameter is μ = 0.005. (TIF) [file pone.0108423.s007.tif]

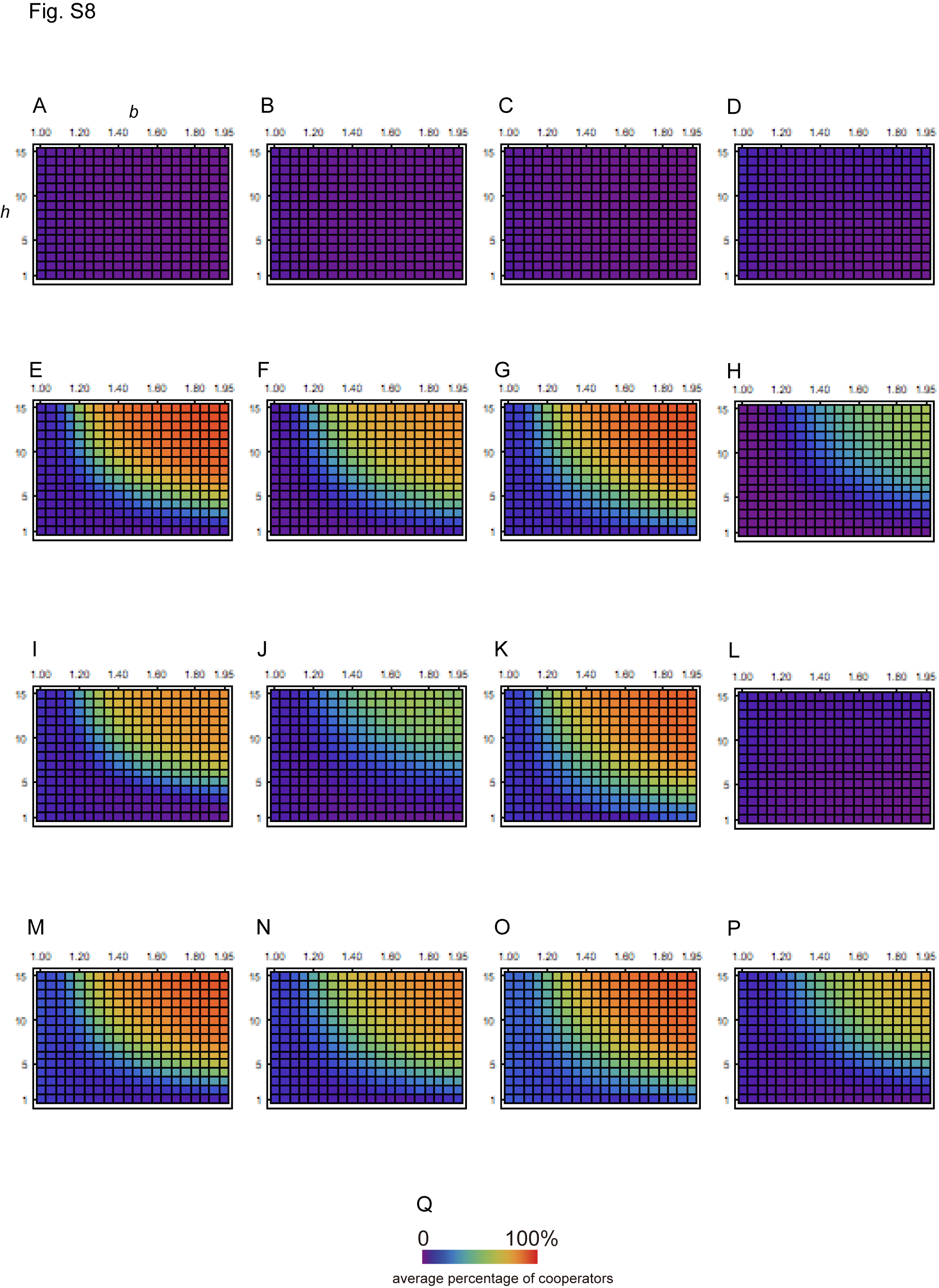

Supplement: Figure S8 — The effect of parameters h and b on simulation outcomes when m = 5 and N = 100. Shown is the average percentage of cooperators in the entire population over 100 runs, in each of which 10,000 generations were simulated. (A−D) are for the participation selection, (E−H) the exclusion selection, (I−L) the same PE selection, and (M−P) the different PE selection. (A, E, I and M) is for the Average criterion, (B, F, J and N) the Median criterion, (C, G, K and O) the Maximum criterion, and (D, H, L and P) the Minimum criterion. (Q) presents the relationship between percentage and color in all graphs. (E) is the same as Fig. 2C. The other parameter is μ = 0.005. (TIF) [file pone.0108423.s008.tif]
